# Effectiveness of a shared decision-making program in reducing unplanned dialysis in advanced chronic kidney disease: a retrospective cohort study

**DOI:** 10.1186/s12882-025-04144-w

**Published:** 2025-05-02

**Authors:** Pannawat Mongkolrattanakul, Kittiphan Chienwichai

**Affiliations:** 1 Division of Nephrology, Department of Internal Medicine, Phanatnikhom Hospital, Chonburi, Thailand; 2 Division of Nephrology, Department of Internal Medicine, Hatyai Hospital, Songkhla, 90110 Thailand

**Keywords:** Shared decision-making, Dialysis, End-stage kidney disease, Renal replacement therapy, Unplanned dialysis, Chronic kidney disease

## Abstract

**Background:**

To evaluate the effectiveness of a Shared Decision-Making (SDM) program in reducing unplanned dialysis among patients with advanced chronic kidney disease (CKD) and to identify factors predictive of unplanned dialysis.

**Methods:**

This retrospective cohort study was conducted at Phanatnikhom Hospital in Chonburi, Thailand, from October 2021 to September 2023. Patients aged 18 years and older with CKD stages 4 and 5 who were receiving renal replacement therapy (RRT) were included. Starting in October 2022, the Shared Decision-Making (SDM) program was implemented as the standard of care. Baseline demographic data, dialysis modalities, and the incidence of unplanned dialysis were collected. Unplanned dialysis was defined as dialysis initiated through a temporary catheter or within a short time frame after the initial dialysis decision.

**Results:**

Among 111 patients, 66 received SDM, and 45 received usual care. The incidence of unplanned dialysis was significantly lower in the SDM group compared to the usual care group (33.3% vs. 66.7%, *p* < 0.001). Multivariate analysis indicated that participation in the SDM program (OR = 0.19, *p* = 0.001), peritoneal dialysis (OR = 0.26, *p* = 0.032), and higher serum albumin at the initiation of dialysis (OR = 0.33, *p* = 0.014) were protective factors against unplanned dialysis.

**Conclusions:**

The SDM program effectively reduces unplanned dialysis in patients with advanced CKD by aligning medical decisions with patient preferences and priorities. Peritoneal dialysis and higher serum albumin levels at dialysis initiation are also associated with lower rates of unplanned dialysis.

## Introduction

End stage kidney disease (ESKD) is a major global health issue that necessitates renal replacement therapy (RRT) for patient survival. In 2010, approximately 2.6 million people worldwide received RRT [1]. This figure is projected to more than double to 5.4 million by 2030, with the most rapid growth expected in Asia [1]. Currently, hemodialysis and peritoneal dialysis are the primary treatment options for patients with ESKD in Thailand.

Studies have shown that starting RRT in an unplanned fashion, which refers to the initiation of RRT without adequate preparation and planning, is associated with morbidity, mortality, and increased healthcare costs [2–6]. Furthermore, planned dialysis initiation is associated with better quality of life [7, 8]. Despite the importance of planning for RRT, 15%-70% of patients initiate therapy in an unplanned manner [9–11]. Although predialysis education is offered to patients and caregivers to minimize unplanned dialysis, its effectiveness remains uncertain due to persistently low awareness and understanding [12, 13]. Additionally, discrepancies between the content of predialysis education and the priorities of patients and their families have been recognized as major factors contributing to delays in timely access creation [14]. Patients and their families often prioritize concerns such as the trade-offs of dialysis— including costs, lifestyle changes, employability, and the potential burden on family members— which can hinder dialysis planning [15–17]. In contrast, clinicians typically emphasize biomedical information, resulting in a misalignment of perspectives.

Shared decision-making (SDM) is the process by which healthcare providers and patients share information and weigh the medical options and patient priorities to decide on medical care that aligns with stated values and preferences [18]. SDM is regarded as the gold standard in clinical practice for promoting patient engagement and activation, empowering individuals to take an active role in their healthcare decision-making processes [19]. SDM approach increases the likelihood that patients will receive treatments that mirror their values, leading to improved health outcomes and greater adherence to prescribed treatments [20]. A previous study demonstrated that SDM improves patient satisfaction with dialysis modality decisions [21], and patients participating in SDM felt that the choice of dialysis modality was truly their own [22]. Furthermore, SDM is associated with better patient survival [23, 24]. While SDM is recommended to guide RRT modality selection [25], there remains a significant lack of data evaluating its effectiveness in reducing unplanned dialysis.

We conducted a retrospective cohort study to assess the effectiveness of SDM as an intervention aimed at reducing unplanned dialysis when compared to physician-directed decision-making in patients with advanced-stage chronic kidney disease (CKD) at Phanatnikhom Hospital in Chonburi. Additionally, we examined predictive factors related to unplanned dialysis in this population.

## Methods

We reviewed electronic medical records to identify all adult patients (aged ≥ 18 years) with kidney failure undergoing RRT (hemodialysis or peritoneal dialysis) at Phanatnikhom Hospital from October 2021 to September 2023. Starting in October 2022, the hospital implemented SDM as standard practice. All patients with CKD stages 4–5 were provided with SDM before initiating RRT. Before October 2022, the dialysis modality selection was determined through physician-directed decision-making. For the entire cohort, we collected baseline demographic data, the type of dialysis modality chosen, and whether patients underwent unplanned dialysis or participated in the SDM program.

All patients in the cohort received standardized information every six months about the benefits of RRT for prolonging life and the advantages and disadvantages of each dialysis modality. To ensure clarity and consistency, trained nurses delivered this information using a combination of video presentations, PowerPoint slides, and demonstration equipment.

This study was approved by the Ethics Committee of the Chonburi Provincial Health Office (CBO Rec 64–096) and conducted in accordance with the principles outlined in the 2013 version of the Declaration of Helsinki (1975). The requirement for informed consent was waived due to the retrospective nature of the study. All patient data were anonymized for analysis.

## CKD clinic

The CKD clinic operates with a multidisciplinary team that includes nephrologists, specialized nurses, pharmacists, dietitians, and social workers, ensuring comprehensive patient care. Patients with CKD stage 4 are followed up every two to four months, while those with CKD stage 5 are monitored every one to three months to assess disease progression and readiness for RRT. During each clinic visit, patient assessments include evaluation of renal function (eGFR), blood pressure control, nutritional status, and management of comorbidities such as diabetes and hypertension.

## SDM program

Our SDM program aligns with the SHARE approach as outlined by the Agency for Healthcare Research and Quality (AHRQ) [26]. The SHARE approach consists of five key components: (1) Seeking the patient’s participation, (2) Helping the patient explore and compare treatment options, (3) Assessing the patient’s values and preferences, (4) Reaching a decision with the patient, and (5) Evaluating the patient’s decision. Additionally, we incorporate the Best Case/Worst Case framework [27] as a patient decision aid, enabling patients to make informed choices about RRT options, the timing of vascular access or Tenckhoff catheter placement, and the initiation of dialysis based on their individual health preferences and concerns.

**Fig. 1:**
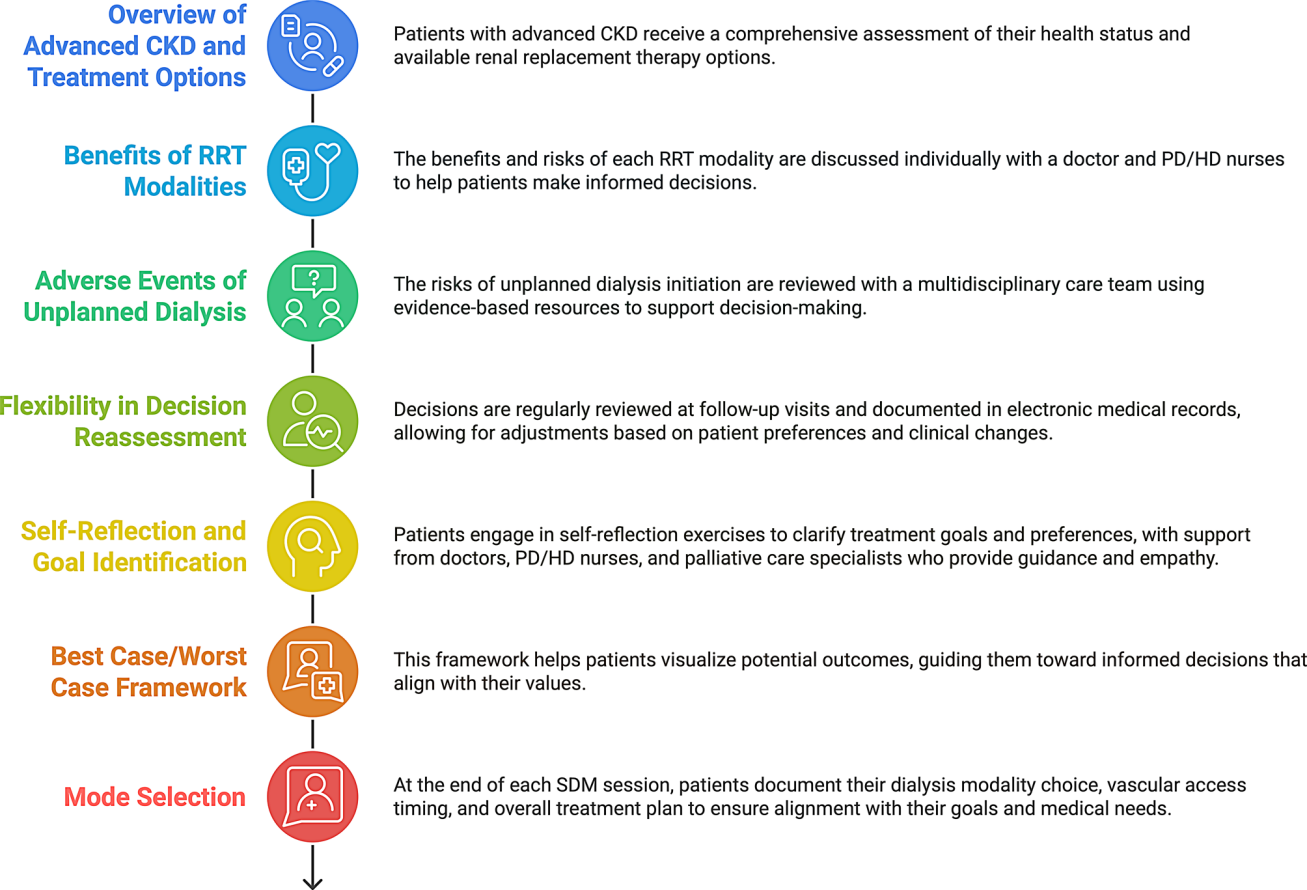
Shared decision-making (SDM) process. This figure illustrates the SDM process for patients with advanced CKD, guiding them through treatment options, personalized discussions, multimedia education, and evidence-based resources to understand RRT benefits, risks, and unplanned dialysis complications. Decisions are regularly reassessed, documented, and adapted to align with patient values. The Best Case/Worst Case framework supports informed choices, ensuring a patient-centered treatment plan, including modality selection and dialysis access planning. *Abbreviations* CKD, Chronic kidney disease; HD, Hemodialysis; PD, Peritoneal dialysis; RRT, Renal replacement therapy; SDM, Shared decision-making

A nephrologist and a trained nurse deliver the SDM program to patients with advanced-stage CKD. It covers seven key topics (Fig. 1): (1) an overview of advanced CKD and available treatment options; (2) the benefits and drawbacks of each RRT modality; (3) potential adverse events linked to unplanned dialysis initiation; (4) flexibility in revising decisions regarding RRT options; (5) self-reflection exercises to help patients identify their values and goals; (6) the Best Case/Worst Case framework; and (7) at the end of each SDM session, patients document their individual values, preferences, and decisions concerning dialysis modalities, the timing of vascular access or Tenckhoff catheter placement, and their overall treatment plan. For patients who have reached a decision, their choices are reviewed at each follow-up visit. If they express uncertainty or wish to rethink their options, the SDM process is revisited. For those unable to decide during the initial session, a follow-up appointment is scheduled for the following month to review the SDM process.

## Outcomes

The primary outcome was the percentage of patients who commenced unplanned dialysis. A nephrologist, unaware of the SDM program’s implementation, reviewed each patient record to assess and confirm whether the criteria for unplanned dialysis were met. Unplanned dialysis is defined as:

- ESKD patients initiate RRT using a temporary dialysis catheter when beginning dialysis [28].
- Patients initiating peritoneal dialysis within 7 days after Tenckhoff catheter insertion [29].

## Statistical analysis

Baseline characteristics were summarized with descriptive statistics, including frequencies and percentages. Differences in proportions between groups were tested using the Chi-square test and Fisher’s exact test as appropriate. Both logistic regression and propensity score–weighted analyses were used to evaluate the impact of the SDM program on unplanned dialysis. Logistic regression was also used to identify predictive factors for unplanned dialysis. Variables included in the multivariate analysis were sex, age, SDM program participation, covariates with *p* < 0.1 in univariate analysis, and key risk factors identified in a recent meta-analysis, including diabetes mellitus, cardiovascular disease, cancer, late referral (defined as nephrology consultation <3 months before dialysis initiation), and hypoalbuminemia [10]. Propensity scores were estimated using generalized boosted models via the WeightIt package [30], and the inverse probability of treatment weighting method was applied to estimate the average treatment effect while adjusting for confounding. Statistical significance was determined at a two-sided 5% level (*p* < 0.05). Data were analyzed using the statistical software package R (R Core Team, 2024) [31].

Based on our data, which indicates an incidence of unplanned dialysis at 65% before the intervention and an expected reduction to 35% after implementing the SDM program, we calculated a required sample size of 86 patients [32]. This sample size ensures the study has 80% power to detect the effectiveness of the SDM program, using a two-sided alpha level of 0.05.

## Results

A total of 116 patients met our inclusion criteria; however, 5 patients were excluded due to incomplete baseline demographic and laboratory data. Table 1 summarizes the clinical characteristics of 111 patients with kidney failure undergoing RRT. Baseline characteristics were comparable between the usual care (*n* = 45) and SDM (*n* = 66) groups regarding sex, age, employment status, and residence. SDM patients reported significantly higher income levels, with 16.67% versus 4.44% earning 5,000–10,000 baht and 15.15% versus 4.44% earning over 10,000 baht (*p* = 0.016). Comorbidities such as diabetes mellitus (75.56% vs. 63.64%), cardiovascular disease (31.11% vs. 33.33%), and cancer (2.22% vs. 4.54%) did not show significant differences between groups. Although not statistically significant, SDM patients tended to have a lower BMI (median 23.09 vs. 25.22, *p* = 0.089) and were more likely to receive peritoneal dialysis (27.27% vs. 13.33%, *p* = 0.129). Among patients receiving hemodialysis, those in the SDM group were significantly more likely to receive preemptive vascular access than those in the usual care group (60.42% vs. 34.15%, *p* = 0.024).

**Table 1:** Clinical characteristics of the patients with end stage kidney disease undergoing renal replacement therapy (*n* = 111)

Variables	Usual care (*n* = 45)	Receive SDM (*n* = 66)	*P* value
Male (%)	24 (53.33)	37 (56.06)	0.929
Age			0.695
≤60 year (%)	23 (51.11)	30 (45.45)	
>60 year (%)	22 (48.89)	36 (54.55)	
BMI (kg/m^2^)	25.22 (22.41–27.58)	23.09 (19.05–26.80)	0.089
Employment status			0.977
Employed (%)	12 (26.67)	19 (28.79)	
Unemployed (%)	33 (73.33)	47 (71.21)	
Education level			0.464
Illiterate (%)	1 (2.22)	6 (9.09)	
Elementary school (%)	30 (66.67)	37 (56.06)	
High school (%)	12 (26.67)	20 (30.30)	
College or above (%)	2 (4.44)	3 (4.54)	
Active smoker (%)	13 (28.89)	20 (30.30)	1.000
Income (per month)			0.016
<5,000 baths (%)	41 (91.11)	45 (68.18)	
5,000–10,000 baths (%)	2 (4.44)	11 (16.67)	
>10,000 baths (%)	2 (4.44)	10 (15.15)	
Residence			1.000
Rural (%)	32 (71.11)	46 (69.70)	
Urban (%)	13 (28.89)	20 (30.30)	
Comorbidities			
Diabetes mellitus (%)	34 (75.56)	42 (63.64)	0.263
Hypertension (%)	40 (88.89)	59 (89.39)	1.000
Cardiovascular disease (%)	14 (31.11)	22 (33.33)	0.969
Stroke (%)	4 (8.89)	9 (13.64)	0.555
Cancer (%)	1 (2.22)	3 (4.54)	0.645
Time referral (days)	223 (57–375)	108 (5–397)	0.353
Dialysis modalities			0.129
Hemodialysis (%)	39 (86.67)	48 (72.72)	
Peritoneal dialysis (%)	6 (13.33)	18 (27.27)	

*Note* Values are presented as mean ± standard deviation, median (interquartile range), or number (%)

*Abbreviations* BMI, body mass index; CKD, chronic kidney disease; SDM, Shared Decision-Making

Table 2 presents biochemical variables from 3 to 6 months prior to dialysis initiation and at the time of dialysis initiation. No significant differences were observed between the two groups in BUN, serum creatinine, eGFR, serum sodium, serum potassium, serum bicarbonate, or serum albumin 3–6 months before dialysis initiation. At dialysis initiation, patients in the SDM group displayed significantly higher BUN levels than those in the usual care group (102.86 mg/dL vs. 85.78 mg/dL, *p* = 0.021). However, there were no significant differences in serum creatinine, eGFR, or other biochemical variables between the two groups.

**Table 2:** Biochemical variables 3–6 months before and during dialysis initiation in patients receiving usual care and SDM (*n* = 111)

Variables	Usual Care (*n* = 45)	Receive SDM (*n* = 66)	*P* value
**At 3–6 months before dialysis initiation**			
BUN (mg/dL)	56.50 (43.00–78.75)	59.01 (41.00–72.50)	0.764
Serum creatinine (mg/dL)	5.38 (3.37–7.84)	4.72 (3.73–5.88)	0.275
eGFR (ml/min/1.73 m^2^)	9.53 (6.26–13.30)	11.30 (7.88–16.80)	0.201
Serum sodium (mEq/L)	137 (136–139)	138 (136–140)	0.635
Serum potassium (mEq/L)	4.40 (4.12–4.90)	4.20 (3.80–4.60)	0.102
Serum bicarbonate (mEq/L)	23 (21–24)	23 (21–25)	0.400
Serum albumin (g/dL)	3.05 (2.40–3.40)	2.90 (2.40–3.40)	0.832
**At dialysis initiation**			
BUN (mg/dL)	85.78 ± 39.33	102.86 ± 35.13	0.021
Serum creatinine (mg/dL)	8.24 (6.61–12.50)	9.02 (7.41–12.30)	0.534
eGFR (ml/min/1.73 m^2^)	5.40 (3.66–7.60)	4.94 (3.81–6.22)	0.698
Serum sodium (mEq/L)	136 (133–138)	136 (133–139)	0.573
Serum potassium (mEq/L)	4.10 (3.60–4.57)	4.10 (3.60–4.50)	0.988
Serum bicarbonate (mEq/L)	21.22 ± 5.97	21.00 ± 5.29	0.841
Serum albumin (g/dL)	2.71 ± 0.65	2.64 ± 0.59	0.584

*Note* Values are presented as mean ± standard deviation, median (interquartile range)

*Abbreviations* BUN, blood urea nitrogen; eGFR, estimated glomerular filtration rate; SDM, Shared Decision-Making

## Comparison of unplanned dialysis incidence between patients who receive usual care and SDM program

Table 3 illustrates the impact of the SDM program on the incidence of unplanned dialysis among patients with ESKD receiving RRT. Among patients receiving standard care, 66.67% experienced unplanned dialysis. In contrast, following the introduction of the SDM program, the percentage of patients undergoing unplanned dialysis significantly decreased to 33.33%. After adjusting for potential confounding factors, including sex, age, diabetes mellitus, cardiovascular disease, cancer, late referral, dialysis modality, serum bicarbonate, and serum albumin at the time of dialysis initiation, patients who participated in the SDM program demonstrated significantly lower odds of requiring unplanned dialysis compared to those receiving standard care. The adjusted odds ratio was 0.19 (95% CI: 0.07–0.47; *p* = 0.001). In propensity score–weighted analyses, patients who participated in the SDM were associated with significantly lower odds of requiring unplanned dialysis (OR, 0.19; 95% CI, 0.10–0.36; *p* < 0.001) compared with those receiving standard care.

**Table 3:** Comparison of the proportion of unplanned dialysis in patients with end stage kidney disease undergoing renal replacement therapy before and after implementing the Shared Decision-Making program

Primary endpoint	Usual care (*n* = 45)	Receive SDM (*n* = 66)	Multivariate analysis odds ratio (95% CI)^†^	*P* value	Propensity score–weighted analysis odds ratio (95% CI)^‡^	*P* value
	number (percent)					
Unplanned dialysis	30 (66.67%)	22 (33.33%)	0.19 (0.07–0.47)	0.001	0.19 (0.10–0.36)	<0.001

^†^ The odds ratios with 95% confidence intervals and P-values were calculated using a logistic regression model to compare patients receiving the SDM program with those receiving usual care, adjusting for sex, age, diabetes mellitus, cardiovascular disease, cancer, late referral dialysis modality, serum bicarbonate, and serum albumin at dialysis initiation

^‡^ Propensity score–weighted analysis was conducted using generalized boosted models to estimate propensity score weights, accounting for potential confounders in the two groups

*Abbreviations* CKD, chronic kidney disease; CI, confidence interval; SDM, Shared Decision-Making

## The predictive factor for unplanned dialysis

Table 4 presents the univariate and multivariate logistic regression analyses of clinical factors associated with unplanned dialysis. In the univariate analysis, peritoneal dialysis was significantly linked to a reduced likelihood of unplanned dialysis compared to hemodialysis (OR = 0.30; 95% CI: 0.10–0.78; *p* = 0.019). Serum albumin levels at the initiation of dialysis were inversely correlated with the occurrence of unplanned dialysis (OR = 0.47; 95% CI: 0.23–0.89; *p* = 0.026), and participation in the SDM program was identified as a significant protective factor (OR = 0.25; 95% CI: 0.11–0.55; *p* = 0.001).

**Table 4:** Univariate and multivariate logistic regression analyses of clinical factors for unplanned dialysis

	Univariate analysis			Multivariate analysis		
	OR	95% CI	*P* value	OR	95% CI	*P* value
Male sex	1.23	0.58–2.62	0.586	1.06	0.42–2.64	0.905
Age						
≤ 60 year (ref.)	1					
> 60 year	0.63	0.30–1.33	0.228	0.50	0.19–1.31	0.163
BMI (per kg/m^2^)	1.03	0.96–1.10	0.419			
Employment status						
Employed (ref.)	1					
Unemployed	1.10	0.48–2.55	0.825			
Education						
Illiterate (ref.)	1					
Elementary school	1.14	0.24–6.20	0.863			
High school	1.33	0.25–7.70	0.732			
College or above	0.89	0.08–9.47	0.921			
Active smoker	1.55	0.69–3.56	0.292			
Income (per month)						
<5,000 baths (ref.)	1					
5,000–10,000 baths	2.02	0.62–7.16	0.249			
>10,000 baths	1.26	0.37–4.34	0.705			
Residence						
Urban (ref.)						
Rural	0.77	0.34–1.73	0.522			
Hypertension	0.60	0.17–1.99	0.402			
Diabetes mellitus	1.07	0.48–2.40	0.871	0.60	0.21–1.62	0.318
Cardiovascular disease	1.02	0.46–2.27	0.956	1.10	0.41–3.01	0.843
Stroke	0.97	0.29–3.12	0.958			
Cancer	0.37	0.02–2.96	0.391	0.66	0.03–6.84	0.751
Late referral^†^	1.07	0.50–2.28	0.862	1.48	0.55–4.12	0.441
Dialysis modality						
In-center hemodialysis (ref.)	1					
Peritoneal dialysis	0.30	0.10–0.78	0.019	0.26	0.07–0.85	0.032
At 3–6 months before dialysis initiation						
BUN (per mg/dL)	1.01	0.99–1.03	0.254			
Serum creatinine (per mg/dL)	1.09	0.94–1.29	0.258			
Serum sodium (per mEq/L)	1.05	0.92–1.22	0.451			
Serum potassium (per mEq/L)	1.14	0.64–2.05	0.648			
Serum bicarbonate (per mEq/L)	0.92	0.79–1.05	0.231			
Serum albumin (per g/dL)	0.63	0.32–1.19	0.160			
At dialysis initiation						
BUN (per mg/dL)	1.00	0.99–1.01	0.926			
Serum creatinine (per mg/dL)	1.04	0.97–1.12	0.260			
Serum sodium (per mEq/L)	0.96	0.89–1.03	0.292			
Serum potassium (per mEq/L)	0.92	0.54–1.55	0.761			
Serum bicarbonate (per mEq/L)	0.94	0.88–1.01	0.098	0.96	0.88–1.04	0.340
Serum albumin (per g/dL)	0.47	0.23–0.89	0.026	0.33	0.13–0.77	0.014
Receive SDM or Usual care						
Usual care (ref.)	1					
Receive SDM	0.25	0.11–0.55	0.001	0.19	0.07–0.47	0.001

^†^ Late referral was defined as patients consulting a nephrologist <3 months prior to dialysis initiation

*Abbreviations* BUN, blood urea nitrogen, CKD, chronic kidney disease; OR, odds ratio; ref., reference; CI, confidence interval; SDM, Shared Decision-Making

In the multivariate analysis, peritoneal dialysis remained significantly associated with a lower risk of unplanned dialysis (OR = 0.26; 95% CI: 0.07–0.85; *p* = 0.032). Likewise, serum albumin at dialysis initiation demonstrated a strong negative association with unplanned dialysis (OR = 0.33; 95% CI: 0.13–0.77; *p* = 0.014). Participation in the SDM program was independently linked to a decreased likelihood of unplanned dialysis (OR = 0.19; 95% CI: 0.07–0.47; *p* = 0.001).

## Discussion

This retrospective cohort study demonstrates the effectiveness of the SDM program in significantly reducing the incidence of unplanned dialysis. Identified protective factors against unplanned dialysis include peritoneal dialysis and participation in the SDM program. Additionally, hypoalbuminemia at dialysis initiation was associated with unplanned dialysis. These findings underscore the importance of the SDM program in improving care and outcomes for patients with advanced-stage CKD.

Currently, there are no proven interventions to reduce the incidence of unplanned dialysis effectively. Although numerous studies have identified risk factors associated with unplanned dialysis, most of these factors are unmodifiable, including cardiovascular disease, advanced age, the underlying cause of kidney disease, cancer, and diabetes [10]. Furthermore, evidence suggests that unplanned dialysis often results from intentional decisions rather than delays related to the healthcare system or physicians [33–36]. The intent to delay dialysis frequently stems from a mix of fears and practical concerns, including exaggerated fears of the procedure, pain, financial costs, disruptions to lifestyle or work, and worries about being a burden to their families. Additionally, social influences such as hearsay, family involvement, perceptual and emotional barriers, and the experiences of others play a significant role [14]. Interactions with healthcare providers, including mistrust and interpersonal tensions, further worsen these delays [14].

SDM empowers patients and physicians to collaborate on healthcare decisions by discussing available options, their associated benefits and risks, and considering the patient’s values, preferences, and circumstances [37]. SDM is crucial in complex choices, such as selecting a dialysis modality or determining when to create vascular access. This approach integrates evidence-based information into the consultation process, enabling informed discussions that align medical recommendations with patients’ personal values and preferences. Consequently, SDM effectively addresses patients’ practical concerns, counters the influence of hearsay, and enhances patient-doctor relationships, ultimately contributing to a reduction in the incidence of unplanned dialysis, as demonstrated in our study.

Hypoalbuminemia has been linked to unplanned dialysis [38]. This can occur due to intercurrent infections or inflammation, which may increase protein catabolism, worsen malnutrition, and contribute to kidney injury, thereby accelerating the initiation of dialysis. Alternatively, patients needing unplanned dialysis might postpone treatment for various reasons, leading to uremic symptoms and worsening malnutrition. The lack of an observed association between serum albumin levels 3–6 months prior to dialysis initiation indicates that hypoalbuminemia may develop closer to the time of dialysis initiation, likely driven by acute events rather than long-term trends.

In our study, a higher proportion of patients in the SDM group elected peritoneal dialysis compared to the control group, likely driven by the SDM process that emphasizes individualized discussions about the advantages and disadvantages of each modality. This finding aligns with a previous study indicating that SDM is associated with a greater likelihood of patients choosing peritoneal dialysis over hemodialysis [39–41]. This approach appears to empower patients who value autonomy to opt for peritoneal dialysis, despite its demands for extensive self-care, family involvement, and a supportive home environment. Although this difference did not reach statistical significance, the trend suggests that SDM may better align treatment choices with patient preferences and practical considerations. Furthermore, peritoneal dialysis is associated with a reduction in unplanned dialysis, likely due to the modality’s preparatory requirements that help mitigate the occurrence of unplanned dialysis events.

Our findings revealed that the SDM group had a higher proportion of patients receiving preemptive vascular access compared to the control group, likely due to the SDM program’s emphasis on proactive planning and individualized discussions about the potential benefits and drawbacks of preemptive vascular access. However, we excluded preemptive vascular access from the multivariable regression due to significant collinearity with the SDM variable, as including both could lead to unstable estimates and obscure the independent effect of the SDM program.

The strength of our study lies in its novel finding that SDM can serve as an effective intervention to reduce the incidence of unplanned dialysis. However, our study also has several limitations. First, as a retrospective study, the observed differences in the effects of the SDM program between groups may be confounded and not necessarily causal. For example, although we adjusted for sex, age, diabetes mellitus, cardiovascular disease, cancer, late referral dialysis modality, serum bicarbonate, and serum albumin at dialysis initiation between the SDM program and usual care groups, we could not account for other factors, such as family or social support, health literacy, acute kidney injury, and medication use, which may have contributed to the differences in unplanned dialysis incidence. Additionally, the absence of uniformly or quantitatively recorded clinical manifestations of uremia prevented us from systematically capturing the severity of uremic symptoms, which could have influenced both the decision and the timing for initiating dialysis. Second, our study utilized a historical control group (usual care group), which may introduce potential confounding factors. These include variations in the standard of care over time and differences in the characteristics of the enrolled populations, such as disease severity and overall health status. However, the baseline characteristics, including age and comorbidities, were comparable between the two groups. Moreover, conducting a randomized controlled trial to compare SDM and usual care groups poses challenges, as SDM is currently recognized as the gold standard of care for patients with CKD. Third, it was not feasible to blind the nephrologists and trained nurses administering the SDM program. However, the program strictly adheres to the SHARE approach, as outlined by AHRQ, which helps to minimize co-intervention by ensuring a structured and standardized process. Fourth, the small sample size in our study may have limited our ability to detect statistically significant differences between groups, potentially increasing the risk of Type II errors. Finally, our study was conducted in Thailand, a developing country, which may limit its generalizability to other countries due to differences in patient preferences, values, and healthcare systems.

## Conclusion

In conclusion, our study demonstrates that SDM is an effective intervention for reducing unplanned dialysis, with peritoneal dialysis serving as a protective factor. Future prospective cohort studies are necessary to evaluate the efficacy, feasibility, cost-effectiveness, and other important clinical outcomes—such as dialysis-related complications and mortality—of SDM programs in a larger population of advanced CKD patients to confirm their role as a viable intervention.

## Data Availability

The datasets analyzed during the current study are available from the corresponding author upon reasonable request.

## Abbreviations

AHRQ: Agency for Healthcare Research and Quality
BMI: Body mass index
BUN: Blood urea nitrogen
CKD: Chronic kidney disease
eGFR: Estimated glomerular filtration rate
ESKD: End stage kidney disease
RRT: Renal replacement therapy
SDM: Shared decision-making
